# Fiber-Based SERS-Fluidic Polymeric Platforms for Improved Optical Analysis of Liquids

**DOI:** 10.3390/bioengineering10060676

**Published:** 2023-06-01

**Authors:** Caterina Credi, Caterina Dallari, Sara Nocentini, Gabriele Gatta, Elena Bianchi, Diederik S. Wiersma, Francesco S. Pavone

**Affiliations:** 1 European Laboratory for Non-Linear Spectroscopy (LENS), Via Nello Carrara 1, 50019 Sesto Fiorentino, Italy; dallari@lens.unifi.it (C.D.); s.nocentini@inrim.it (S.N.); gatta@lens.unifi.it (G.G.); diederik.wiersma@unifi.it (D.S.W.); francesco.pavone@unifi.it (F.S.P.); 2 National Institute of Optics, National Research Council (INO-CNR), Via Nello Carrara 1, 50019 Sesto Fiorentino, Italy; 3 Department of Physics, University of Florence, Via G. Sansone 1, 50019 Sesto Fiorentino, Italy; 4 National Institute of Metrology (INRiM), 10135 Turin, Italy; 5 Department of Chemistry, Materials and Chemical Engineering, Politecnico di Milano, Piazza Leonardo da Vinci 32, 20133 Milano, Italy; elena1.bianchi@polimi.it

**Keywords:** surface-enhanced Raman spectroscopy (SERS), nanoparticles, microfluidics, 3D printing, lab-on-chip (LOC), liquid samples, photocurable perfluoropolyethers

## Abstract

Downsizing surface-enhanced Raman spectroscopy (SERS) within microfluidic devices has opened interesting perspectives for the development of low-cost and portable (bio)sensors for the optical analysis of liquid samples. Despite the research efforts, SERS-fluidic devices still rely either on the use of expensive bulky set-ups or on polymeric devices giving spurious background signals fabricated via expensive manufacturing processes. Here, polymeric platforms integrating fluidics and optics were fabricated with versatile designs allowing easy coupling with fiber-based Raman systems. For the first time, anti-fouling photocurable perfluoropolyether (PFPE) was explored for high-throughput SERS-integrating chip fabrication via replica molding of negative stamps obtained through standard and advanced fabrication processes. The PFPE devices comprised networks of channels for fluid handling and for optical fiber housing with multiple orientations. Embedded microfeatures were used to control the relative positioning of the fibers, thus guaranteeing the highest signal delivering and collection. The feasibility of PFPE devices as fiber-based SERS fluidic platforms was demonstrated through the straightforward acquisition of Raman-SERS spectra of a mixture of gold nanoparticles as SERS substrates with rhodamine 6G (Rh6G) at decreasing concentrations. In the presence of high-performing gold nanostars, the Rh6G signal was detectable at dilutions down to the nanomolar level even without tight focusing and working at low laser power—a key aspect for analyte detection in real-world biomedical and environmental applications.

## 1. Introduction

To date, optical detection techniques have gained increasing attention for the development of sensors based on the non-destructive analysis of liquid samples with potential applications in the clinic–diagnostic field, to precociously reveal pathological biomarkers in biofluids, as well as in the environmental and agrifood sectors to reveal contaminants [1]. In recent decades, the technological advances in nanomaterials and nanotechnologies have been applied to the development of optical sensors, exploiting metal plasmonic nanostructures such as gold nanoparticles (NPs), which are used as optical transducers due to their unique combination of physical and chemical properties [2,3]. Gold NPs can be synthesized with fast and low-cost processes with varied morphology that can be engineered to match the wavelength of irradiation, thus ensuring extraordinary efficacy in adsorbing and scattering light [4]. In the development of the sensors, gold NPs also play the functional role of the recognition element of the sensor by selectively capturing the target analytes of interest. To this end, the gold surfaces can be modified through the physical or covalent self-assembling of linker molecules to increase the NPs’ affinity towards the targets with respect to the overall heterogeneous matrix, thus increasing the sensitivity of the systems. The synergic combination of these attributes has been smartly approached to develop surface-enhanced Raman spectroscopy (SERS) assays, which exploit the unique phenomenon of surface plasmons to selectively amplify the Raman signal of target molecules adsorbed on the surfaces of NPs irradiated by a laser source. It has been reported that SERS-based assays maintain their high sensitivity even when measurements are performed directly on liquids, avoiding sample drying steps that could invalidate the measurements since these can induce conformational changes of the analytes to be targeted [5]. However, uncontrolled evaporation phenomena on the microscopic scale could occur during SERS measurements when irradiating the liquids dropped on the sample holder (commonly calcium fluoride slices). In addition, sample handling and mixing with NPs could be critical steps due to the potential contamination of the samples when working in realistic scenarios outside laboratories. Additionally, the use of bulky Raman equipment does not facilitate addressing the lab–clinic transfer. To overcome these limitations and to guarantee the high specificity and reproducibility of NP-based SERS, recent approaches are focused on downsizing SERS technology within lab-on-chip (LoC) devices, monolithic functional platforms comprising micron-sized channels designed for both a reduced volume of fluids and light manipulation on the micrometric scale, thus showing optimal size matching between microchannels, the optical beams and the biological elements to be targeted. LoCs can host functional microfluidic components, including in-built micromixers guaranteeing efficient NPs–samples mixing and/or separators enabling multiplexing analysis, avoiding cross contamination [6,7]. In light of the above considerations, the hyphenation of SERS and microfluidics represents a win–win approach [8,9,10,11,12]. In addition, as already reported for Raman-microfluidic, SERS-LoCs can be coupled with fiber-based Raman systems characterized by higher compactness and portability. Waveguides and optical fibers can be housed directly in the LoCs to precisely deliver and collect the optical signals at the detection chamber level, avoiding the use of confocal microscopes or complex supporting optical elements for sample relative alignment with the set-up [13,14]. Undoubtedly, these technological changes could reduce the complexity and the size of the standard Raman set-up used in the laboratories. However, this size reduction relies on the exploitation of hand-held spectrometers whose performances are limited by the poor quantum yield in the NIR region. Furthermore, to increase the scattered Raman signal they require to work at higher laser powerthat can damage biological samples [15]. To address this issue, excitation and detection optical fibers can be embedded in the devices with different design/configurations [13,16], which, in turn, require many engineering steps as well as expensive and long-lasting lithography-based fabrication processes. Furthermore, they are based on NP aggregation phenomena, which could require long irradiation steps before performing the measurements, thus affecting their repeatability [17,18,19,20]. Another constraint is represented by the materials that are typically used for microfluidics, which can introduce Raman background spurious signals, especially when working with polymers (e.g., polydimethylsiloxane, PDMS) and glass [21]. Currently, quartz represents the best alternative for Raman-microfluidic systems, but with high costs for processing and microstructuring.

As a step forward, in this work, we present the development of a novel SERS-fluidic platform for improved analysis of liquid samples characterized by ease of design and fabrication as well as ease of coupling with a fiber-based Raman set-up avoiding complex optics and optomechanical components. Starting from previous results obtained by our group in the rapid prototyping of PDMS-based LoCs for SERS applications, we explored the use of photocurable perfluoropolyethers (PFPEs) to engineer advanced polymeric devices exhibiting suitable optical properties (such as transparency) and negligible Raman signal. The latter is a fundamental advancement for improving the limit of detection of analytes based on SERS monitoring. By combining advanced and traditional micro-fabrication processes based on photo-polymerization, PFPE LoCs can be rapidly fabricated with different designs aimed at identifying the configuration for a simple-to-operate chip guaranteeing the highest sensitivity upon coupling with the Raman set-up. In addition, the high thermal, chemical and ageing stability of PFPEs combined with their resistance to aspecific protein surface adsorption represent the optimal combination for re-usable devices [22,23,24]. This aspect is crucial for measurement repeatability, considering that SERS functionality is achieved by mixing liquid samples with gold NPs whose SERS performances are strictly related to the NPs’ colloidal stability, which can be altered by boundary conditions. Different NPs’ morphologies were also explored and, finally, the best SERS-fluidic devices successfully enabled the detection of low-concentration analytes even at low laser power. By synergically combining multifuncional LoCs obtained by PFPEs micro-structuring and signal enhancers gold NPs, we showed the advantages of this compact novel polymeric platform for improved liquid analysis.

## 2. Materials and Methods

Materials: Gold(III) chloride trihydrate (HAuCl4 ∙ 3H_2_O), trisodium citrate dihydrate (C_6_H_5_O_7_Na_3_ ∙ 2H_2_O), L-(+)-ascorbic acid (AA), silver nitrate (AgNO_3_), hydrochloric acid (HCl), nitric acid (HNO_3_—70%), (±)–α-Lipoic acid (LA), N-(3-Dimethylaminopropyl)-N-ethylcarbodiimide hydrochloride (EDC), N-Hydroxysuccinimide (NHS), (3-Aminopropyl) triethoxysilane (APTES), Rhodamine 6 G (Rh6 G) and hydrogen peroxide solution (30% in H_2_O) are purchased from Merck (Darmstadt, Germany) and used without further purification. PDMS Sylgard 184 silicone elastomer is purchased from Dow Corning (Midland, MI, USA). Vitra 430^®^ stereolithographic photoresist is purchased from Digital Wax^®^ Systems (DWS^®^, Vicenza, Italy). A bifunctional PFPE urethane dimethacrylate oligomer with molecular weight Mn = 1980 g/mol (PFPE-DMA) is purchased from ACOTA—Specialty material solutions (commercial name Fluorolink ™ MD700), and the photoinitiator hydroxyl-2-methyl-1-phenyl-propan-1-one (Darocur(R) 1173, Ciba) is purchased from Merck (Darmstadt, Germany).

Synthesis and characterization of gold NPs: Gold nanoparticles (AuNPs) are synthesized through the seeded-growth process described by Yuan et al. [25]. The seed solution of 15 nm nanospheres (NSps) is prepared by adding 1.5 mL of 30 mM HAuCl_4_ ∙ 3H_2_O (1%) to 48.5 mL of boiling and stirring MilliQ (stirring 7 position, 250 °C) in a sterile 50 mL glass flask. After 10 s, 4.5 mL of 38.8 mM sodium citrate solution is added to the solution. The solution is stirred under heating for 15 min, and then stirred without heating for 30 min. For nanostar (NSt) synthesis, 0.083 mL of 30 mM HAuCl_4_ ∙ 3H_2_O (1% solution) is added to 9.917 mL of MilliQ in 50 mL Falcon. Next, 60 μL of 1M HCl and 100 μL of the NSps solution are added to the solution. Then, 100 μL of 2 mM AgNO_3_ and 50 μL of 0.1 M ascorbic acid are added simultaneously. The solution is stirred for 30–60 s, while its color turns from light red to dark grey (or blue). Immediately afterwards, NSts are centrifuged for 20 min at 2500 rpm in a 1 mL Eppendorf^®^ flask and redispersed in 100 μL of ultrapure water. The plasmonic resonant properties of NPs colloidal solutions are characterized in the range from 400 nm to 850 nm with a UV-Vis-NIR spectrophotometer (Lambda 950 instrument, Perkin Elmer, Waltham, MA, USA). UV WinLab Software (v 6.4.0) is used to acquire spectra, and data are processed with Origin software. The hydrodynamic dimensions and the polydispersity are acquired through dynamic light scattering (DLS) analysis performed with a Malvern Zetasizer Nano series ZS90. Measurements are performed with a fixed scattering angle of 90° at 25 °C. Each sample is measured three times, and each measurement consists of about 30 acquisitions. Cumulating statistics are used to measure the hydrodynamic diameter and polydispersity. Data are then processed with Origin software. The structural features of the NPs are characterized using transmission electron microscopy (TEM, CM 12 PHILIPS, Philips, Netherlands, Europe).

PFPEs LoCs fabrication: Polymeric circuits were fabricated through the replica molding (REM) of negative stamps that were fabricated through 3D printing with stereolithography (SLA) and standard lithography. For SLA printing, computer-aided design (CAD) models of polymeric negative molds (approximately 25 mm × 35 mm) were designed using ‘SolidWorks’ software (Dassault Systèmes, Vélizy-Villacoublay, France). The 3D models were first processed with parametric software Nauta+^®^ (DWS systems), which allows the object to eventually be reoriented on the working platform and then loaded into Fictor^®^ (DWS, systems) for numerical slicing according to the user-imposed building parameters. The laser speed ranged between 250 and 5000 mm s^−1^, with the layer thickness between 20 μm and 100 μm. Finally, physical molds were built with bench-top XFAB 2500 HD stereolithography apparatus (DWS systems) equipped with a monochromatic actinic laser source (Solid State Bluedge^®^ BE-1500A/BE-1500AHR) with an emitting output power of 30 mW at λ = 405 nm and a 50 μm laser spot diameter. At the end of the printing process, the SLA-structured molds were washed in ethanol to remove unreacted resin and then post-cured for 15 min in a dedicated ultraviolet curing unit (λ = 405 nm, Model S Ultraviolet Curing Unit, DWS^®^) to accomplish the total polymer conversion. Negative molds prepared using standard lithography were fabricated in a clean room facility (PoliFAB—Politecnico di Milano) through the photopolymerization of a three-layer mold made of SU-8 (Kayaku Advanced Materials, Westborough, MA 01581, USA). A thin layer of SU-8 2005 was spun for coating at 3000 rpm, prebaked, cured and post-baked on a 2″ Si wafer to obtain a flat 5 nm thick base for the further monolithic mold structure. On this base, a first layer of SU8-2100 was spun for coating at 2500 rpm and prebaked (65 °C 5 min, 95 °C 25 min). The wafer was then transferred to an MLA100 (Heidelberg Maskless aligner, λ = 365 nm, resolution down to 1 µm, depending on aspect ratio and shape), where the geometrical patterns of optofluidic channels were written on the SU-8 layer. A second SU-8 2100 layer was then spun for coating at 2500 rpm, prebaked as the first one was (acting as a post-bake for the previous patterned layer) and re-transferred to the MLA 100, where the fluidic channel pattern was written and aligned to the first one. The wafer underwent a post-bake treatment (65 °C 5 min, 95 °C 12 min) before being developed for about 30 min, in immersion, in the SU-8 developer (2-methoxy-1-methylethyl acetate), washed in acetone then isopropyl alcohol and dried with a nitrogen gun. The structures were then observed under an optical microscope and measures checked with an optical profiler (Filmetrics Profilm3D, Filmetrics, Unterhaching, Germany).

As reported later, depending on the fiber-based Raman set-up used to acquire spectra, PFPE devices of varied designs could be rapidly prepared. The key elements of the devices were the network of channels used to load the liquid NP mixture to be analyzed and the network of channels used to house optical fibers for light delivery and collection within the circuits. The “fluidic” network comprised microchannels of rectangular-shaped cross-sections with a 2 mm width and 200 µm height. The “optical” network comprised microchannels of square-shaped cross-sections of 100 μm both in width and in height for the excitation path and 200 μm for the collection path. Devices with 45°, 90° and 175° relative angles between the irradiating and collecting fibers were fabricated and compared for coupling efficiency (Figure S1). To fabricate the devices, PFPE-DMA was mixed with PI (photoinitiator) at 2% with respect to the total weight of the resin (wt), poured onto the molds and UV-irradiated for 50 s (λ = 365 nm, 37 mW) under nitrogen, corresponding to 95% of conversion. Thanks to the low surface tension characterizing the fluorinated material, the PFPE-DMA microstructured replica was easily released from the mold. Sealed devices were realized by coupling the PFPE-DMA structured replica with a 200 µm thick flat layer of PFPE-DMA UV-exposed for 30 s (corresponding to a kinetic conversion of 80%), and further UV-irradiating the entire structure to seal the layers together via completion of the polymerization process [26].

**Figure 1:**
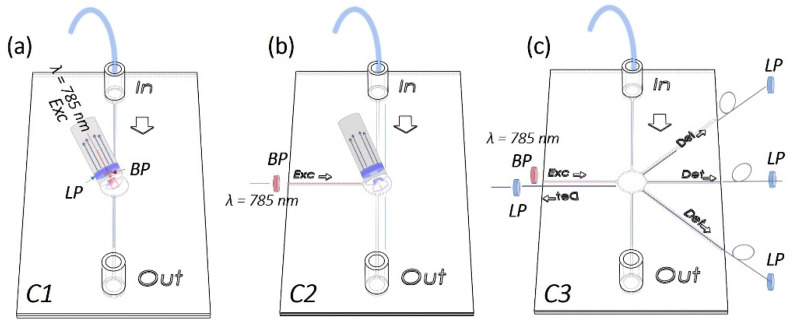
Scheme representing the configurations adopted for smart coupling of PFPE devices with fiber-based Raman systems operating with different irradiation collection paths. (**a**) In configuration 1, the distal probe of the customized Raman set-up is vertically aligned to the fluidic channel and used for light delivery and collection through the PFPE walls. (**b**) In configuration 2, the distal probe is used to collect the signal, while the laser light is delivered straightforwardly/directly to the sample through a 100 µm optical fiber coupled with the laser and embedded in the “optical” network of the devices. (**c**) In configuration 3, single optical fibers rearranged with different relative angles are inserted in the fluidic devices for Raman excitation and collection. BP: band-pass, LP: long-pass.

Raman set-up configurations: In order to acquire Raman-SERS spectra, PFPE devices were coupled with fiber-based Raman systems rearranged in three different configurations in terms of number of fibers and relative position between the excitation and detection paths. In more detail, as schematized in Figure 1a, for the SERS-fluidic configuration 1, following the previous results obtained by our group for PDMS devices [27], the “fluidic” channel of the PFPE device was aligned with the distal end of the Raman probe of a customized set-up whose detailed description is reported elsewhere [28]. The probe consists of a metallic jacket holding together 25 optical fibers (100 μm core diameter, low -OH content, 0.22 NA). The central fiber is connected to a power-variable laser diode for light delivering (λ = 785 nm, maximum power in freespace ≈ 350 mW), while the other 24 fibers, which are used for collecting the signal in backward configuration, are connected to the detection system comprising a monochromator (microHR HORIBA Scientific Edicon, NJ, USA) and a −60 °C cooled CCD array camera (Syncerity HORIBA Scientific, Edicon, NJ, US). A band-pass filter and a coaxial ring-shaped long-pass filter are placed on the free tip of the bundle in order to clean the laser line to block the unwanted fluorescence generated inside the excitation fiber and Rayleigh-scattered photons. In the SERS-fluidic configuration 2 (Figure 1b), the distal end of the probe was kept aligned to the “fluidic” network and was used for collecting the signal as for configuration 1, while the 100 µm optical fiber (Avantes, P100SMA) for the straightforward delivery of laser light to the fluid was embedded in the PFPE device via simple insertion in the “optical” network. In this configuration, a commercial notch filter at 785 nm (LL01-785-12.5, MaxLine Laser Filter, Semrock, Rochester, NY, USA) is inserted in the irradiation path through a dedicated holder (Avantes, Variable In-Line Filter Holder, FH-INL) equipped with two SMA connectors for serial connection with the laser output. In the SERS-fluidic configuration 3 (Figure 1c), the Raman signal was excited as in configuration 2 and collected using one 200 µm low-OH commercial optical fiber inserted in the device with different relative angles with respect to the irradiation fiber. The collecting fiber was coupled to a spectrometer (Andor, iDus 416, 830 L/mm, Oxford Instrument, Abingdon, UK) equipped with a back-illuminated and thermoelectrically cooled CCD camera (Kymera 193). A standard long-pass filter (LP02–785RE-12.5 Semrock, Rochester, NY, USA) was inserted in series with a second holder (Avantes, Variable In-Line Filter Holder, FH-INL) to filter Rayleigh-scattered photons.

SERS-fluidic performances: The performances of SERS-fluidic devices coupled with a fiber-based Raman set-up with different irradiation collection configurations were tested by mixing one volume of rhodamine 6G (Rh6G) aqueous solution at varied concentrations (from 1 mM to 10^−9^ mM depending on the SERS-fluidic configuration adopted) with one volume of gold NSps (13 nM) or one volume of AuNSts (8 nM) injected into the microfluidic chip from a dedicated inlet and immediately recording the spectra. Depending on the Raman set-up configuration, the laser power ranged between 150 mW and 5 mW, the acquisition time was 20 s and the measurements were repeated 5 times for spectral averaging. In order to extract the Raman signal of interest, fluorescence and background signals were subtracted from the acquired raw spectra through the Vancouver Raman Algorithm, a dedicated software package for automatic autofluorescence background subtraction for Raman spectroscopy [29]. Data were further analyzed with Origin software. For peak assignment, the Raman spectrum of Rh6G powder was taken into account (Figure S2).

## 3. Results

Monolithic PFPE devices smartly integrating a network of channels for fluidic and optics functionalities were fabricated with different designs in terms of channel shape, size and relative position between the “fluidic” and the “optical” paths to enable simple interfacing with portable fiber-based Raman systems while guaranteeing optimal signal coupling efficiency. As depicted in the scheme in Figure 1, SERS-fluidic platforms were developed and tested for straightforward liquid sensing in three different configurations: coupling PFPE fluidic devices with a hand-held Raman probe used for light delivering and collection (configuration 1); coupling PFPE optofluidic devices with a hand-held probe for light collection while light is delivered through an embedded optical fiber (configuration 2); embedding optical fibers for light delivery and collection in the PFPE optofluidic devices (configuration 3). For sake of brevity, from here on, the configurations will be named C1, C2 and C3.

**Figure 2:**
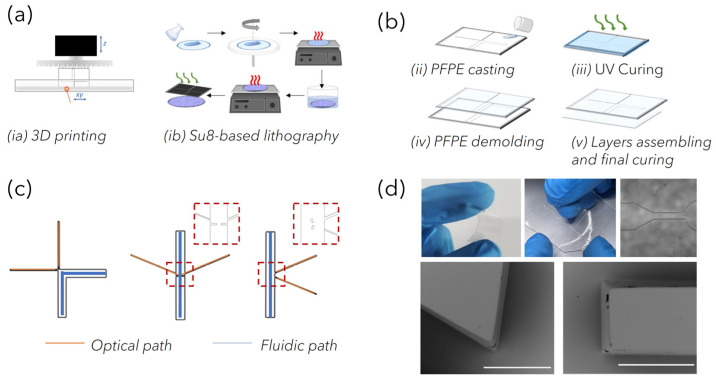
(**a**) Fabrication of negative molds through (ia) laser-based 3D printing and (ib) Su8-based lithography. (**b**) Scheme depicting the REM fabrication of monolithic PFPE devices: (ii) pouring PFPE prepolymer-PI mixture onto the molds, (iii) UV-curing PFPE, (iv) peeling off the mold and (v) sealing with a flat layer of partially cured PFPE. (**c**) Designs of the optofluidic devices fabricated with different relative positions between the optical and fluidic paths. (**d**) Pictures (upper panel) and SEM images (lower panel) of monolithic PFPE microfluidic devices obtained by implementing the replica molding of 3D printed stamps (the scale bar for SEM images is 200 µm).

**Figure 3:**
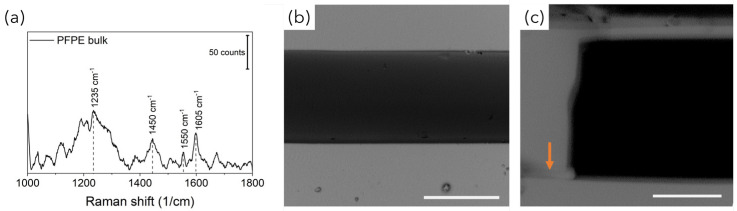
(**a**) Raman spectrum of PFPE. SEM images of the cross section of PFPE monolithic devices demonstrating that neither (**b**) clogging/warping phenomena nor (**c**) layer lamination were observed (the arrow indicates the layer interface). Scalebar is 100 µm in all cases.

To this end, a strategy combining advanced and traditional manufacturing processes, previously used by our group with PDMS [26] and characterized by high flexibility, was optimized for the rapid prototyping of customized opto-fluidic devices with high repeatability. PFPE micro-structured layers were obtained through the REM of plastic negative molds fabricated by exploiting both advanced and traditional manufacturing technologies (Figure 2a). Indeed, laser-based SLA enabled parallel fast production (<20 min) at low cost of molds for C1 and C2, characterized by features down to 100 µm (corresponding to the accuracy of the bench-top SLA equipment). On the other hand, high-resolution standard SU8-based lithography was used to fabricate devices for C3, whose design incorporates customized microstructures down to 50 µm that were introduced to facilitate the alignment of fibers within the channels, as discussed later. The overall dimensions of the devices (about 25 mm × 35 mm) were considered to correctly position the “fluidic” and “optical” paths and to guarantee easy handling, allowing for routine analysis. Then, depending on the SERS-fluidic configurations adopted, the main skeleton of the devices could include a linear or L-shaped channel (blue path in Figure 2c) with a rectangular-shaped cross section (x in w and x in h) used for fluid loading, intersected by one (for C2) or more linear channels (for C3) used to house the optical fibers (orange path in Figure 2c). These optical channels were characterized by squared cross-sections, and their nominal dimension was equal to the nominal core diameter of the inserted fibers to guarantee perfect channel–fiber surface contact and to avoid leakage phenomena. As shown in the MO (microscope optical) images in Figure 2d, optically transparent PFPE replicas were obtained, as expected, with high fidelity. In addition, during the REM fabrication process, the low surface tension typical of fluorinated materials allowed us to easily peel off the PFPE layer from the molds as well as to create many PFPE replicas, avoiding mold degradation as well as the passivation treatments that are often required. At the same time, the use of the UV-based photopolymerization process guarantees fast PFPE curing at room temperature as well, avoiding the thermal treatments that are required for PDMS curing that could warp the 3D-printed plastic molds. Another substantial advantage with respect to PDMS for SERS-fluidic application is represented by the lower Raman spurious signal emitted by PFPE (Figure 3a).

Monolithic PFPE devices were obtained by placing the µ-structured PFPE replica on a flat PFPE replica and further UV-exposing the system to seal the two layers together via completion of the polymerization process. As shown in the SEM images in Figure 3, complete sealing of the PFPE layers was successfully achieved, avoiding clogging or warping phenomena (Figure 3b) as well as lamination at the PFPE layer interfaces (Figure 3c). Additionally, the wavy texture patterning of the PFPE channel surfaces that is typical of layer-by-layer 3D material deposition did not affect the tight sealing of the devices.

Before analyzing the effect on the SERS performances of the optofluidic systems developed with different excitation/detection configurations, we preliminarily demonstrated the feasibility of PFPE-based devices for SERS liquid sampling using C1 and comparing with previous results obtained for PDMS fluidic devices. To this end, Rh6G solution (0.5 mM) was diluted 10, 100, 1000 and 10,000 times with water to obtain decreasing concentration solutions, and then mixed with AuNPs in the ratio 1:1. AuNPs are considered as efficient SERS substrates characterized by high availability at low cost through fast chemical synthesis and the possibility for tailored tuning of their morphology and, thus, of their resonant properties, with absorbance peaks at 520 nm and 680 nm for Au NSps and Au NSts, respectively, with dimensions ranging from 17 nm to 85 nm (Figure S3).

**Figure 4:**
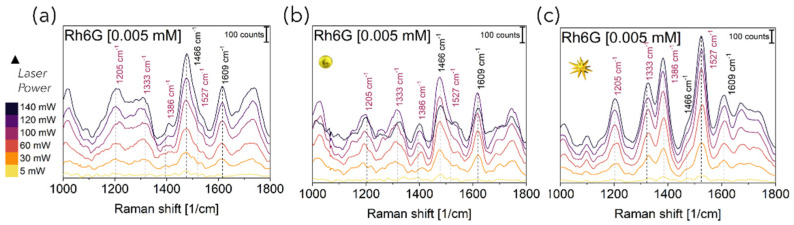
Representative Raman-SERS spectra of Rhodamine 6G aqueous solution at 5 μM concentration, acquired at varied laser power for C1 by aligning the distal end of the Raman fiber with PFPE microfluidic devices (**a**) without NPs (**b**) and upon mixing with Au NSps and (**c**) Au NSts. Wavenumbers were assigned to relative peaks. Black and purple labels were used to mark peaks characteristic of PFPE and Rh6G, respectively. Spectra are shifted vertically for clarity.

These Rh6G-NP mixtures were then fluxed through the fluidic networks and individually detected. Raman spectra were acquired by aligning the distal end of the Raman fiber probe housing both the excitation and collection optical fibers in a backward configuration (C1, Figure 1a). As can be seen from the spectra reported in Figure 4 for Rh6G 5 µM, the same behavior of an increase in the signal intensity upon increasing laser power was observed with and without NPs. The trend was observed for all Rh6G concentrations tested (Figure S4). All spectra reported both narrow peaks characteristic of the device (marked with black labels) and characteristic of the Rh6G (marked with purple labels). Indeed, considering that the cone of irradiation of C1 passes through the PFPE top layer, the Raman peaks around 1460 cm^−1^ and 1610 cm^−1^ could be considered spurious signal from the polymer. This was further confirmed by the fact that the intensity of these peaks did not significantly vary when increasing the Rh6G concentration. Conversely, Rh6G Raman peaks at 1205 cm^−1^ (aromatic ring N–H), 1333 cm^−1^ (CH_3_ umbrella mode), 1386 cm^−1^ (CH_3_–CH_2_ deformation) and 1527 cm^−1^ (aromatic C = C stretching) were seen to appear and to progressively vary in intensity when raising the Rh6G concentration. In more detail, the Raman spectra reported in Figure 4a obtained for PFPE devices without NPs are characterized by strong PFPE peaks with the Rh6G profile slightly visible. Conversely, the effect of SERS was clearly observed for Rh6G mixed with Au NSps (Figure 4b) and even better upon mixing with Au NSts (Figure 4c), considering that Rh6G peaks are still visible for concentrations down to 0.5 µM (see Figure S4 in Supplementary Information), at least one order of magnitude lower with respect to the literature and halved with respect to the C1 configuration working with PDMS devices [27].

**Figure 5:**
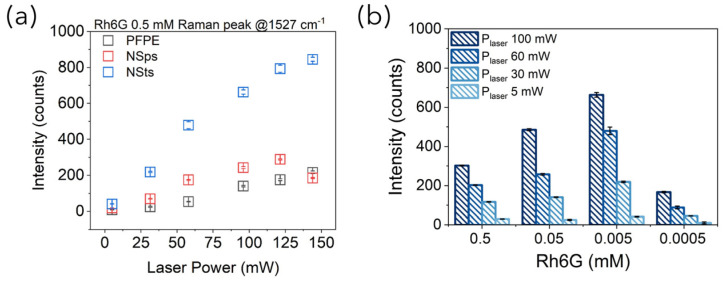
Variation in the Raman peak intensity of (**a**) Rh6G at 1527 cm^−1^ as a function of laser power at Rh6G decreasing concentration with and without nanoparticles. (**b**) Rh6G Raman peak intensity plotted against Rh6G concentration for different laser powers in the presence of gold NSts.

More interestingly, by plotting the intensity of the Raman rhodamine peak at around 1527 cm^−1^ as a function of the laser power, acquired with and without NPs, the SERS enhancement was observed to follow a linear trend with a higher slope for NSts with respect to NSps, thus further attesting to their superior efficiency as SERS substrates (Figure 5a). This means that in the presence of NSts, small increments in laser power can induce higher increments in Raman signal intensity with respect to NSps. This could be crucial for acquiring Raman spectra of those analytes that are characterized by small Raman cross-sections (e.g., biomolecules such as proteins). The same trend was observed at all concentrations tested (Figure S5) and also for the other two Rh6G Raman characteristic peaks at 1333 cm^−1^ and at 1386 cm^−1^ (Figure S6). No SERS enhancement was observed for the intensity of the 1609 cm^−1^ Raman peak of PFPE, neither with NSps nor with NSts and the varied Rh6G tested (Figure S7).

The higher SERS enhancement achieved with AuNSts was ascribable to the anisotropic form of NSts, characterized by sharp tips with a nanosized radius of curvature protruding from the core, which results in intrinsic NSt plasmonic properties in resonance with the wavelength of irradiation while providing a huge number of “hot spots” [30]. The Raman peak intensities’ dependence on the different concentration of Rh6G is reported in Figure 5b using different excitation laser powers. To understand this trend, we measured the UV-vis extinction spectra acquired for NPs-Rh6G solutions at varied concentrations (Figure S7). Upon the addition of rhodamine, the optical properties of Au NPs were altered. A new absorbance peak was observed to appear at around 660 nm for NSps, corresponding to particle clustering triggered by the interaction between the Rh6G molecules and the AuNSps (Figure S8a). Rh6G molecules trapped within NSp aggregates (hot spots) should experience a higher incoming irradiation and, thus, a higher SERS enhancement. Instead, a smaller red-shift of the maximum peak of absorbance from 668 nm to 720 nm is observed for NSts, representing, as expected, optimal SERS enhancers with respect to NSps (Figure S8b). Finally, as is clearly visible in Figure 5b, by using NSts as SERS substrates, the Raman signal of Rh6G is high even for the lowest Rh6G concentration and when lowering the laser power, a crucial acquisition parameter, especially when working on liquid samples such as biological fluids suffering from analyte unfolding or solvent evaporation, potentially affecting the final measurements. The curious trend observed of decreasing Raman signal at Rh6G concentrations higher than 5 µM is due to the fact that above this concentration threshold, the number of Rh6G molecules adsorbing on the NPs is enough to stabilize the colloidal systems, thus decreasing their clustering behavior and hot spot-based SERS effect [31]. Finally, these experimental results attested that the limit of detection (LOD_1_) of SERS-fluidic devices working in the C1 configuration, corresponding to the lowest concentration at which the Rh6G Raman peak was still visible, is 5 µM: at least one order of magnitude lower with respect to the same fluidic devices fabricated with PDMS [27].

**Figure 6:**
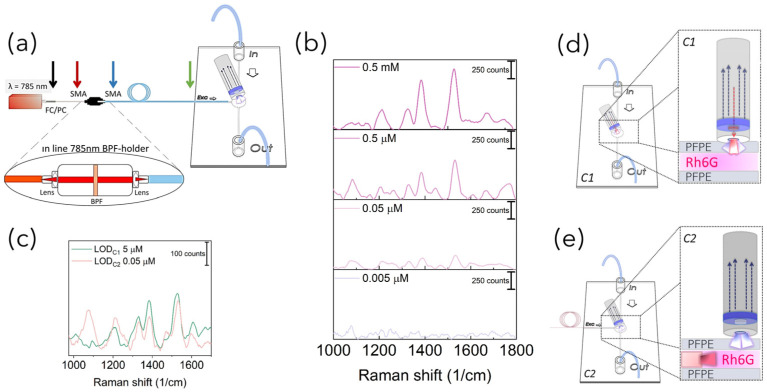
(**a**) Scheme representing the optical path used in configuration C2 to deliver light at the sample level. A filter holder equipped with two lenses was used to insert the BPF in line with the laser. The arrows indicate the interfaces where the laser power was measured to quantify power losses due to set-up changes. (**b**) Representative Raman-SERS spectra acquired in C2 configuration at decreasing Rh6G concentrations upon mixing with AuNSts. C1 vs. C2 (**c**) Raman-SERS spectra obtained at their LOD and (**d**) C1 vs. (**e**) C2 concept schemes of their irradiation collection paths.

Once the feasibility of PFPE devices to work as SERS-fluidic platforms was attested, aiming at further decreasing their LOD, the back-scattering fiber-based Raman set-up coupled with the devices was modified to the C2 configuration by decoupling the excitation and collection part of the probe. To this end, a 100 µm optical fiber used for light irradiation was directly embedded in the devices through simple insertion in a dedicated channel intersecting the “fluidic” network”. The 24 optical fibers held in the C1 configuration were still used to collect the signal. According to the optical scheme reported in Figure 6a, the C2 configuration allowed us to substitute the high-cost custom-sized BPF used to clean the laser wavelength in the C1 Raman probe with a commercial filter mounted serially through a low-cost dedicated holder equipped with two lenses for light collimation. Prior to acquiring the Raman spectra, power losses introduced at the holder–fibers interfaces were characterized, and it transpired that set-up changes in the excitation path with the in-line BPF insertion decreased the laser power by about 80% with respect to its maximum, with values drastically dropping off from 350 mW when in free space down to 50 mW at the output of the fiber (Figure S9). However, previous Raman-SERS spectra obtained upon mixing with Au NSts attested that this point should not represent an issue, considering that high sensitivity could be achieved even for laser power lower than 50 mW. Another limitation connected to the C2 configuration was represented by the spurious fluorescence contribution that could hinder the Raman spectra, which originated from the glassy optical fiber due to an in-plane misalignment between the excitation and detection volumes (Figure S10). However, such a limitation was advantageously exploited to facilitate the relative positioning of the collecting probe with respect to the fiber of irradiation, avoiding the requirement for optical alignment. Indeed, by slightly moving the collecting probe in the XY plane, the optimal position was identified when successfully acquiring Raman-SERS spectra of the Rh6G solution (0.5 mM) mixed with high-performing AuNSts. Then, the LOD of the C2 system (LODc_2_) was also determined through the detection of Rh6G dilutions, and from the Raman peak at 1527 cm^−1^, it was 0.05 µM (Figure 6c), at least two orders of magnitude lower with respect to the LODc_1_.

As schematized in Figure 6d, this could be ascribable to the more efficient delivery of light at the sample level, avoiding irradiation from the outer part of the PFPE device and, thus, decreasing the number of refractive index interfaces changes potentially affecting the number of incoming photons. Furthermore, recorded spectra were free from spurious background signal from the PFPE monolithic device, increasing the signal-to-noise ratio and, therefore, the sensitivity.

To go one step further, and to further simplify the set-up, the millimetric Raman distal probe was completely decoupled to work in the C3 configuration where both the excitation and collection fibers were embedded in the PFPE devices. As previously reported [13], this configuration not only improves the efficiency of incoming light directly towards the detection chamber (as for C2) but also enables the excitation and collection regions to be confined. Another advantage of such confinement is also reflected in the amount of irradiated volume of samples, which is reduced to the area closer to the apex of the optical fibers thus not overlapping with other part of the PFPE devices.

**Figure 7:**
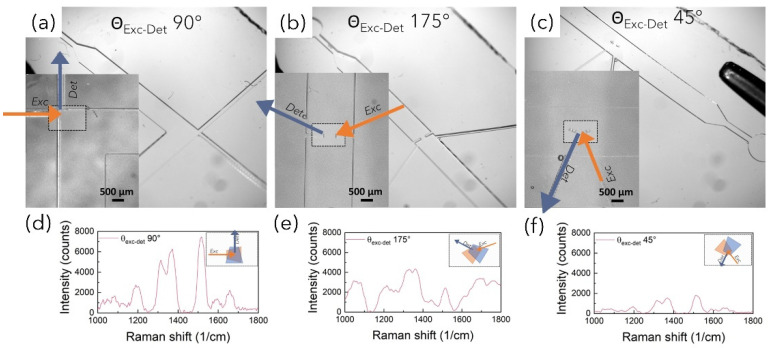
(**a**–**c**) OM images of PFPE optofluidic devices fabricated with varied relative angles between the irradiation and detection fibers (θ_exc_-θ_det_), namely 90°, 75° and 45°. The insets highlight the fluidic–optics intersection areas where simple micron-sized structures have been fabricated to facilitate fiber alignment. The blue and orange arrows represent the irradiation and detection fibers, respectively. (**d**–**f**) Representative Raman-SERS spectra of Rh6G 0.5 mM solutions acquired with the three θ_exc_-θ_det_ upon mixing with Au NSts. The insets show the variation in the Raman signal area collection as a function of θ_exc_-θ_det_.

**Figure 8:**
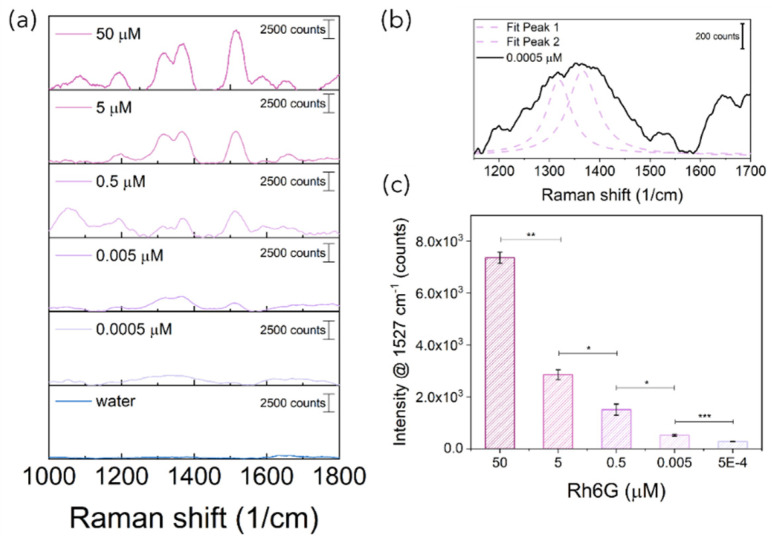
(**a**) Representative Raman-SERS spectra acquired with C3 configurations at decreasing Rh6G concentrations upon mixing with Au NSts. (**b**) SERS spectra and corresponding deconvolution acquired for Rh6G 0.0005 µM mixed with Au NSts; dashed magenta lines are the fitting for the Rh6G characteristic peaks and the continuous black line represents the cumulative fit peaks. (**c**) Mean intensities of the 1527 cm^−1^ Raman peak for decreasing Rh6G concentrations. Each concentration was tested thrice and the signal intensity at Rh6G peak was used to calculate the mean values and the relative deviation standard (DS). Error bars were then calculated as DS divided by the number of samples analyzed (n = 3). NS > 0.05; * *p* < 0.05; ** *p* < 0.01; *** *p* < 0.001; an unpaired Student’s *t*-test was performed.

As shown in the OM images reported in Figure 7a–c, to explore the effect on SERS performances, PFPE optofluidic platforms were fabricated with varied relative angles between the irradiation and detection fibers (θ_exc_-θ_det_), namely 90°, 175° and 45°. To achieve the desired configurations, PFPE devices were designed with the “optical” path for fibers housing differently rearranged with respect to the “fluidic” path where simple micron-sized structures were fabricated for the 175° and 45° configurations to help with the optimal positioning and alignment of fibers, avoiding expensive optics and optomechanics components, hence making it appealing for real-world applications (inset in Figure 7b,c and Figure S11). No additional references were needed for the 90° configuration where the fiber coupling was achieved simply by exploiting the geometry of the embedded waveguides (inset in Figure 7a). After fiber insertion, Rh6G solution (0.5 mM) was mixed with AuNSts, and Raman-SERS spectra were immediately acquired for the three configurations. All recorded spectra were free from background spurious signals originating from the devices with higher intensity (about 4 times), while spectra acquired with the 90° configuration (Figure 7d) presented higher spectral resolution with respect to the 175° (Figure 7e) and 45° configurations (Figure 7f). As graphically represented by the 2D projections reported in the inset in Figure 7d–f, this is likely due to the larger Raman signal collection area as a consequence of the larger overlapping volume of the cone of excitation with the cone of detection. At acute or obtuse angles, the shifting of the collection volumes can either reduce the number of inelastic scattered photons collected or increase the fluorescent background hindering the Raman signal. Hence, the minimum limit of detection for the 90° configuration 3 (LODc_3_) was quantified by acquiring Rh6G aqueous solution at decreasing concentrations upon mixing with AuNSts (Figure 8a). Despite the lower laser power (50 mW) and the number of collecting fibers being drastically decreased from 24 for the C1 and C2 configurations to 1 for the C3 current configuration, Raman peaks ascribable to Rh6G were still visible at concentrations down to 0.0005 µM, at least two orders of magnitude lower than LODc_2_ and four orders of magnitude lower than LODc_1_ (Figure 8b). Furthermore, the single fiber collection configuration enabled us to appreciate significative differences in Raman intensities even at low concentrations (*p* < 0.001), as highlighted with *t*-test results (Figure 8c).

## 4. Conclusions

In the present work, we reported on the robust fabrication/engineering of novel polymeric SERS-fluidic devices whose versatility in terms of channel design simplifies the chip’s connection with portable fiber-based Raman systems, avoiding complex optics and optomechanics components. By combining advanced and traditional micro-fabrication processes based on photo-polymerization, optically transparent monolithic devices were entirely fabricated within 30 min with a UV-curable PFPE whose anti-fouling properties combined with the negligible spurious background Raman signal ensured improved sensitivity with respect to traditional PDMS devices. Additionally, the PFPE replica capability and PFPE replica fidelity guarantees the high-throughput low-cost production of devices from the same 3D-printed or micro-machined molds, thus representing an approach easily adaptable to various microfluidic platforms. Here, PFPE devices were realized with varied geometry in terms of the network of milli-sized channels with embedded micron-sized features where fluidic and optical functionalities could be smartly rearranged, aiming at identifying the optical configuration to achieve the highest efficiency in terms of light delivering and signal collection. Finally, SERS-fluidic devices were successfully exploited to attest their capabilities for on-chip SERS analyses of liquid samples with high repeatability upon interfacing with fiber-based Raman systems. The use of Au NSts as SERS substrates mixed with the sample solutions enabled the rapid detection down to concentrations of 5 nm, avoiding drying steps and tight focusing of high laser power, which eventually affect molecules’ conformation—a crucial aspect for the real-world applications envisioned for PFPE SERS-fluidic systems.

## Data Availability

Not applicable.

## Supplementary Materials

The following supporting information can be downloaded at: https://www.mdpi.com/article/10.3390/bioengineering10060676/s1, Figure S1: CAD schemes of the polymeric devices obtained with (a) 90°, (b) 175° and (c) 45° relative angles between the irradiating and collecting fibers; Figure S2: Raman spectra for Rh6G powder; Figure S3: TEM images for gold (a) nanospheres and (b) nanostars. (c) Size distribution and zeta potential values for Au NSps and Au NSts stabilized with citrate molecules. UV-vis absorption spectra of colloidal solution of gold (d) nanospheres and (e) nanostars; Figure S4: Representative Raman-SERS spectra of Rhodamine 6G aqueous solution at concentrations ranging from 0.5 mM to 0.5 μM, acquired by aligning the distal end of the Raman fiber with PFPE microfluidic devices (a, b, c and d) upon mixing with Au NSps (e, f, g and h) and Au NSts (i, j, k and l). Wavenumbers were assigned to relative peaks. Spectra are shifted vertically for clarity; Figure S5: Variation in the Raman peak intensity of (a–d) Rh6G at 1527 cm^−1^ as a function of laser power at Rh6G decreasing concentration with and without nanoparticles; Figure S6: Variation in the Raman peak intensity of (a–d) Rh6G at 1333 cm^−1^ and (e–h) at 1386 cm^−1^ as a function of laser power at Rh6G decreasing concentration with Au NSps and Au NSts; Figure S7: Variation in the Raman peak intensity of (a–d) of PFPE at 1609 cm^−1^ as a function of laser power at Rh6G decreasing concentration with and without nanoparticles; Figure S8: UV-vis absorption spectra of Rh6G mixed at varied concentrations with (a) Au NSps and (b) Au Nsts. The addition of Rh6G triggers the NP aggregation, resulting in the appearance of a red-shifted peak for NSps and in a slight shift for NSts; Figure S9: Laser power measured at the different interfaces in the irradiation path used for the C2 configuration; Figure S10: (a) Scheme representing the in-plane misalignment between the excitation Raman probe and the glassy optical fiber resulting in (b) spurious fluorescence contribution that could hinder the Raman spectra; Figure S11: OM images of the SU8 molds realized for the (a) 90°, (b) 175°, and (c) 45° configurations.
