# Supplementary material for: Fiber-Based SERS-Fluidic Polymeric Platforms for Improved Optical Analysis of Liquids
Source: Bioengineering (Basel). 2023 Jun 1;10(6):676. doi: 10.3390/bioengineering10060676 (PMC10295140; doi:10.3390/bioengineering10060676)
Supplement: Supplementary file 1 [file bioengineering-10-00676-s001.zip › bioengineering-2398982-supplementary.pdf]

Article

Fiber-Based SERS-Fluidic Polymeric Platforms for Improved Optical Analysis of Liquids

Caterina Credi [1,2,*], Caterina Dallari [1,3], Sara Nocentini [1,2,4], Gabriele Gatta [1], Elena Bianchi [5], Diederik S. Wiersma [1,3,4] and Francesco S. Pavone [1,2,3]

1 European Laboratory for Non-Linear Spectroscopy (LENS), Via Nello Carrara 1, 50019 Sesto Fiorentino, Italy; dallari@lens.unifi.it (C.D.); s.nocentini@inrim.it (S.N.); gatta@lens.unifi.it (G.G.); diederik.wiersma@unifi.it (D.S.W.); francesco.pavone@unifi.it (F.S.P.)
2 National Institute of Optics, National Research Council (INO-CNR), Via Nello Carrara 1, 50019 Sesto Fiorentino, Italy
3 Department of Physics, University of Florence, Via G. Sansone 1, 50019 Sesto Fiorentino, Italy
4 National Institute of Metrology (INRiM), 10135 Turin, Italy
5 Department of Chemistry, Materials and Chemical Engineering, Politecnico di Milano, Piazza Leonardo da Vinci 32, 20133 Milano, Italy; elena1.bianchi@polimi.it
* Correspondence: caterina.credi@gmail.com or caterina.credi@ino.cnr.it; Tel.: +39-055-4572506

Supporting Information

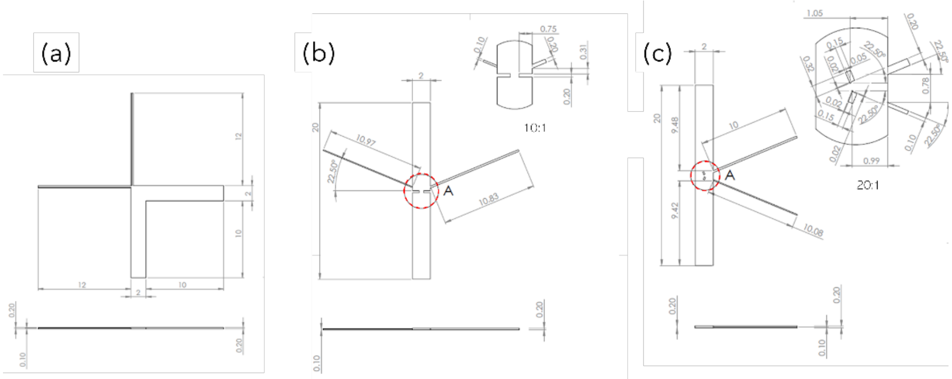

Figure S1. CAD schemes of the polymeric devices obtained with (**a**) 90°, (**b**) 175°, and (c) 45° relative angles between the irradiating and collecting fibers.

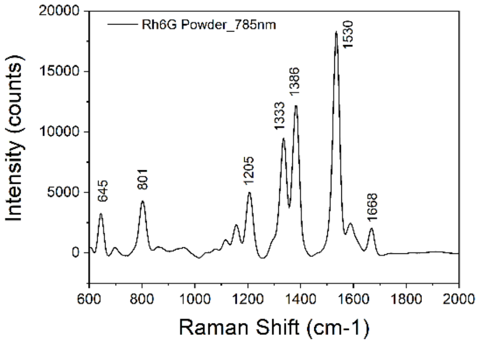

Figure S2. Raman spectra for Rh6G powder.

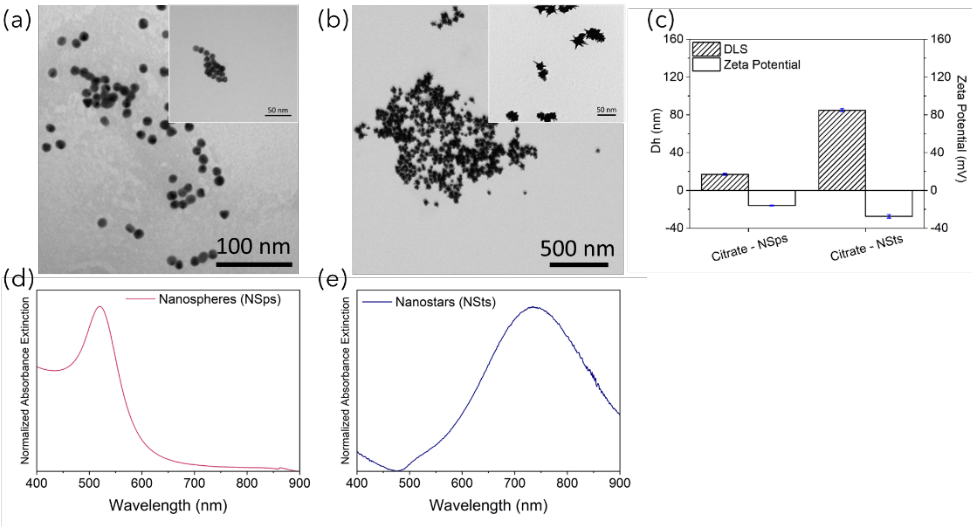

Figure S3. TEM images for gold (**a**) nanospheres (NSps) and (**b**) nanostars (NSts). (**c**) Size distribution and zeta potential values for Au NSps and Au NSts stabilized with citrate molecules. UV-vis absorption spectra of colloidal solution of gold (**d**) nanospheres and (**e**) nanostars.

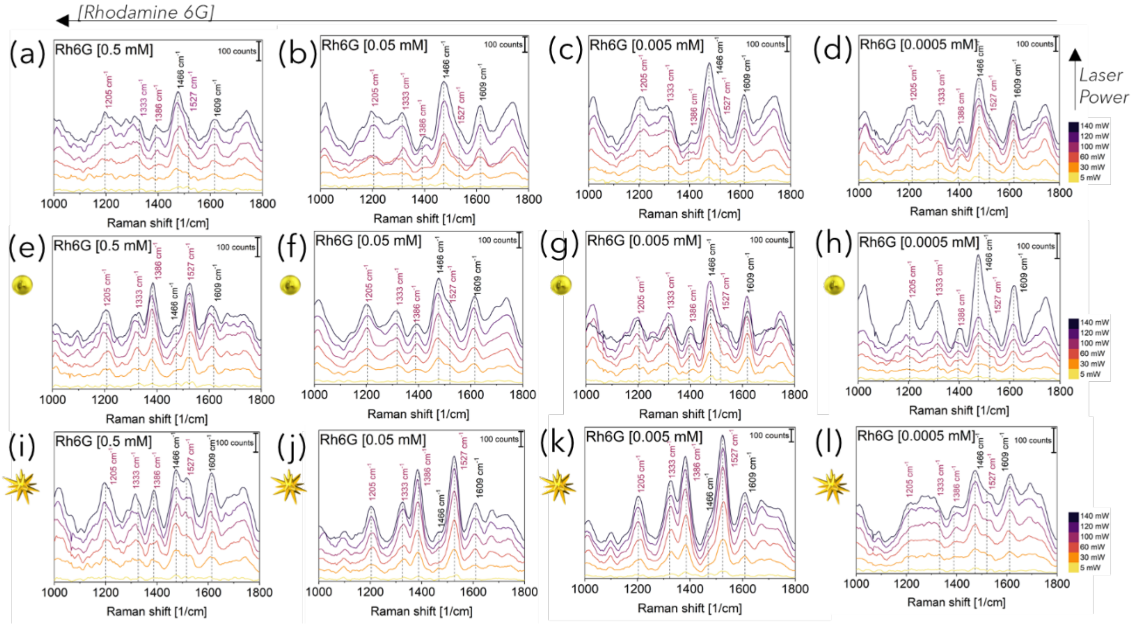

Figure S4. Representative Raman-SERS spectra of Rhodamine 6G aqueous solutions at concentration ranging from 0.5 mM to 0.5 μM, acquired aligning the distal end of the Raman fiber with PFPE microfluidic devices (**a**, **b**, **c**, and **d**), upon mixing with Au NSps (**e**, **f**, **g**, and **h**) and Au NSts (**i**, **j**, **k**, and **l**). Wavenumbers were assigned to relative peaks. Spectra are shifted vertically for clarity.

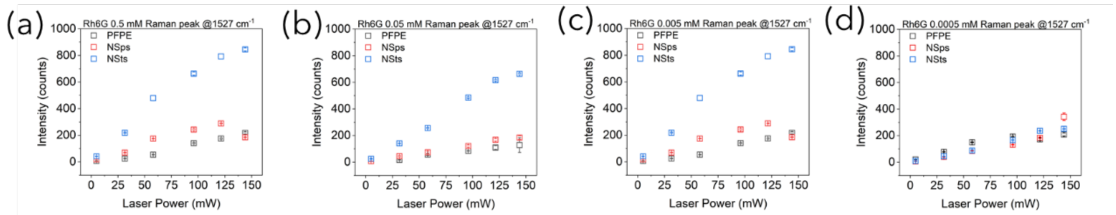

Figure S5. Variation of the Raman peak intensity of (**a**–**d**) Rh6G at 1527 cm^{-1} as function of laser power at Rh6G decreasing concentration without nanoparticles (black squares), with nanospheres (red squares) and with nanostars (blue squares).

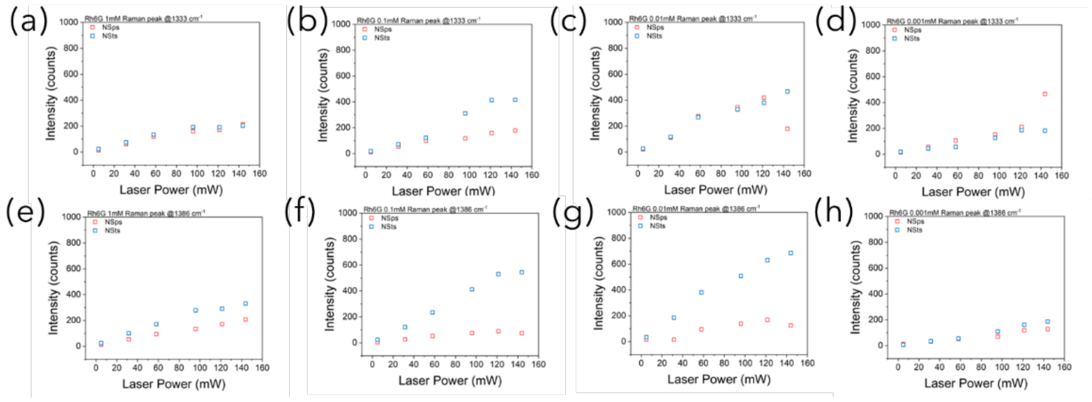

Figure S6. Variation of the Raman peak intensity of Rh6G (**a**–**d**) at 1333 cm^{-1} and (**e-h**) at 1386 cm^{-1} as a function of laser power at Rh6G decreasing concentration with Au NSps (red squares) and Au NSts (blue squares)

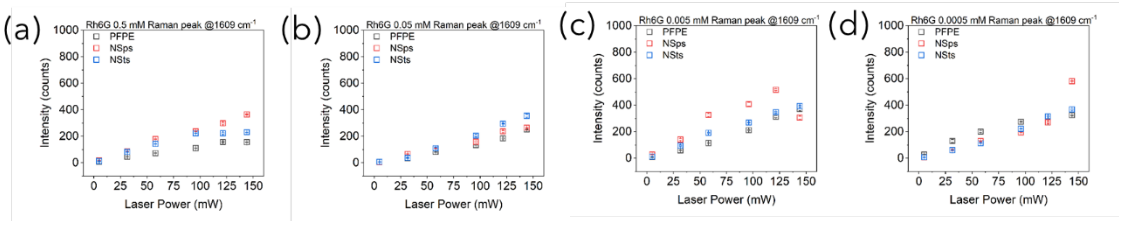

Figure S7. Variation of the Raman peak intensity of (**a**–**d**) of PFPE at 1609 cm^{-1} as function of laser power at Rh6G decreasing concentration with and without nanoparticles.

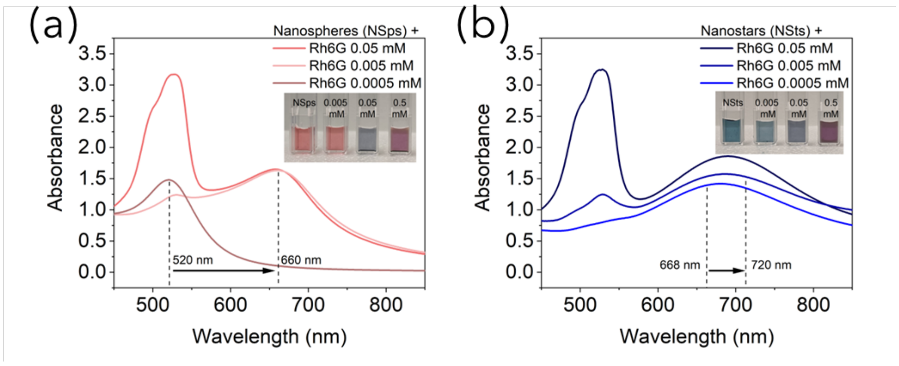

Figure S8. UV-vis absorption spectra of Rh6G mixed at varied concentrations with (**a**) Au NSps and (**b**) Au Nsts. The addition of Rh6G triggers the NPs aggregation resulting in the appearance of a red-shifted peak for NSps and in a slight shift for NSts.

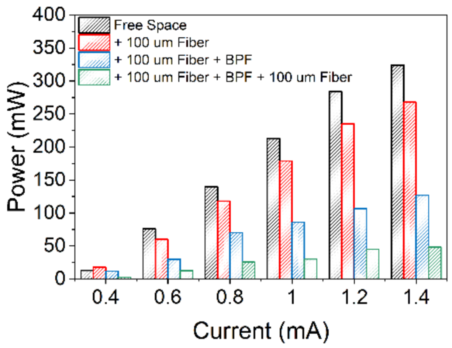

Figure S9. Laser power measured at the different interfaces in the irradiation path used for the C2 configuration.

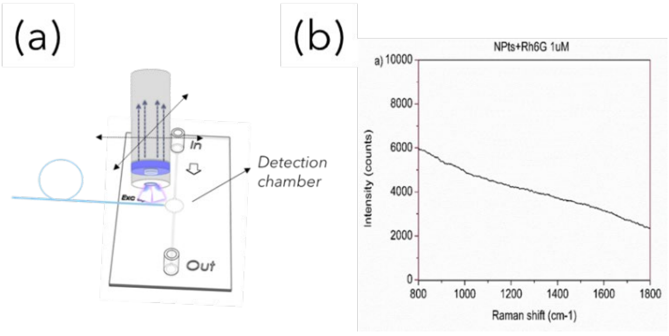

Figure S10. (**a**) Scheme representing the in-plane misalignment between the excitation Raman probe and the glassy optical fiber resulting in (**b**) spurious fluorescence contribution that could hinder the Raman spectra.

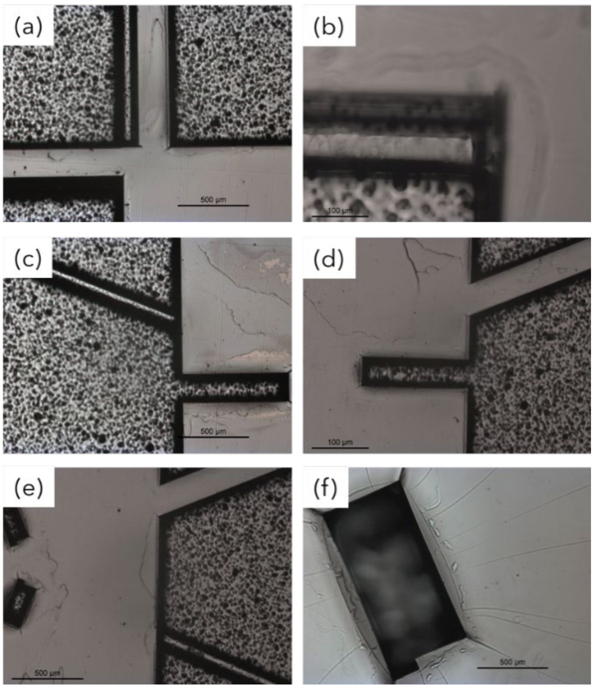

Figure S11. OM images of the SU8 molds realized for the (**a**,**b**) 90°, (**c**,**d**) 175°, and (**e**,**f**) 45° configurations.
